# Exploring social cognition in patients with apathy following acquired brain damage

**DOI:** 10.1186/1471-2377-14-18

**Published:** 2014-01-23

**Authors:** Progress Njomboro, Glyn W Humphreys, Shoumitro Deb

**Affiliations:** 1 Department of Psychology, University of Cape Town, Rondebosch 7701, South Africa; 2 School of Psychology, University of Birmingham, Birmingham, UK; 3 Division of Neurosciences, Imperial College, London, UK

**Keywords:** Apathy, Brain damage, Social cognition, Theory of mind, Emotion perception, Social awareness, Moral reasoning

## Abstract

**Background:**

Research on cognition in apathy has largely focused on executive functions. To the best of our knowledge, no studies have investigated the relationship between apathy symptoms and processes involved in social cognition. Apathy symptoms include attenuated emotional behaviour, low social engagement and social withdrawal, all of which may be linked to underlying socio-cognitive deficits.

**Methods:**

We compared patients with brain damage who also had apathy symptoms against similar patients with brain damage but without apathy symptoms. Both patient groups were also compared against normal controls on key socio-cognitive measures involving moral reasoning, social awareness related to making judgements between normative and non-normative behaviour, Theory of Mind processing, and the perception of facial expressions of emotion. We also controlled for the likely effects of executive deficits and depressive symptoms on these comparisons.

**Results:**

Our results indicated that patients with apathy were distinctively impaired in making moral reasoning decisions and in judging the social appropriateness of behaviour. Deficits in Theory of Mind and perception of facial expressions of emotion did not distinguish patients with apathy from those without apathy.

**Conclusion:**

Our findings point to a possible socio-cognitive profile for apathy symptoms and provide initial insights into how socio-cognitive deficits in patients with apathy may affect social functioning.

## Background

Over the past decade clinical observations and research have helped establish the position of apathy or lack of motivated activity as a common neuropsychiatric disorder following acquired brain damage, with important predictive relations to rehabilitation success [1-3]. Studies in the area have investigated the prevalence of apathy in various neuropsychiatric populations [4], its relationship with other disorders such as depression [5-8], and its association with executive processes involved in the cognitive control of goal-directed activity [9-14]. To the best of our knowledge no studies have investigated how apathy symptoms may relate to deficits in socio-cognitive domains such as social decision making related to moral reasoning and social awareness, Theory of Mind (ToM) judgements, or the perception of facial expressions of emotion. The absence of studies in this area is striking when considered in the context of the functional impairments that patients with apathy show in their day-to-day lives. Behavioural problems characterised by social withdrawal, disinterest in social interactions, and deficits related to the experience and expression of emotions, form a significant part of the clinical picture in apathy [15-18].

Neurological models of motivated behaviour highlight the role of distinct frontal-subcortical circuits in human goal directed activity [11-13,19,20]. Damage to these circuits is thought to underlie apathy symptoms [14,21], and these symptoms have been reported in patients with acquired brain damage involving both frontal and subcortical lesions. For instance, in one meta-analytical study [14] it was found that across five studies, an average of 61% of patients with focal frontal lesions and 41% of patients with subcortical damage presented with apathy symptoms. See also [22,23] for similar findings. Apathy is also often associated with anterior cingulate cortex (ACC) lesions [24-26]. Large or bilateral frontal and ACC lesions are known to cause even more severe apathy [27,28]. Imaging studies have found a relationship between apathy severity and bilateral atrophy of the medial and orbital frontal cortices, the ACC, temporal cortex and medial thalamic regions [18,29-32].

Damage to some of the frontal and subcortical brain areas implicated in apathy is often closely associated with social cognition deficits such as those involving moral judgements, feelings of regret, empathy, and embarrassment [33-36]. In some neuropsychiatric samples, functional and structural disruptions to neural mechanisms sub-serving socio-cognitive processes are thought to underlie significant behavioural aspects of brain pathology [37]. For example, impaired capacities to infer other people’s beliefs and mental states have been reported in a wide range of neuropsychiatric disorders, including autism [38-40], frontal damage [41,42], schizophrenia [43-48], various types of dementias [49-51], antisocial personality disorder [52], and bipolar affective disorder [53]. It is likely that these socio-cognitive deficits make it difficult for patients to cope with and respond appropriately to their social world, resulting in social withdrawal and loss of interest in social life [38,54]. Such lack of interest and social withdrawal are some of the defining features of apathy [17,55].

Some evidence on the neural substrates of social cognition, particularly on the role of emotion processing and its influences on human motivated behaviour has also been forthcoming [56-61]. Most of these studies suggest the involvement of subconscious emotional signals that help initiate and pre-bias goal-directed social behaviour towards adaptive choices [61-63]. It is possible that deficits in the mechanisms that generate these signals may underlie apathy related socio-cognitive dysfunction. The ventromedial prefrontal cortex is thought to play a crucial role in generating these signals [57], and has also been implicated in apathy [64]. Furthermore, patients with apathy show distinct deficits in their ability to initiate or sustain goal directed social interactions [13].

This study focuses on the relationship between apathy symptoms and social cognition. We hypothesized that patients with acquired brain damage who present with apathy symptoms would also present with significant impairments on a battery of socio-cognitive tasks designed to assess moral reasoning, social awareness, ToM, and emotion perception when compared to similar patients without apathy symptoms and also to neurologically intact participants. Our aim was to take patients with brain damage from a wide range of aetiologies in order to give a broad sample of possible relations between apathy and socio-cognitive processes. Measures of executive functioning and depressive symptoms were also taken in order to separate the possible effects of executive deficits and depression on social cognition from those of apathy symptoms. The literature suggests that executive deficits and depressive symptoms are associated with both apathy and some aspects of social cognition dysfunction [10,11,65]. We included 3 executive measures in order to accommodate cognitive deficits across patients. For instance, administering the Stroop test only would have meant dropping patients with language problems. In doing this we tried to obtain at least one executive function score for each of the participants. We however did not take these separate scores as interchangeable. We treated each of the executive function scores as a distinct covariate in our analyses.

## Methods

### Participants

Patients with brain damage were recruited from clinics and rehabilitation centres within the West Midlands region of England. All the patients were assessed at least 6 months post injury. In total 49 patients with neurological damage (see Table 1) and 56 neurologically intact adult controls participated in the study. Imaging results and lesion location data were available in 46 patients (see Table 2). Control participants were recruited from within the same area as patients through local adverts and were screened for neurological and psychiatric illnesses. Different controls took part on different tests. Table 3 gives the demographic characteristics of all participants (including controls) across the individual tests. All participants gave informed written consent. Ethics approval for the study was granted by the Birmingham and Solihull Research Ethics Committee.

**Table 1:** Patient characteristics and cause of brain injury

***Etiology***	***N***	***Sex***	***Age***
		***M = Male***	***Mean & Std Dev***
		***F = Female***	
Cerebrovascular accident (CVA)	24	M = 15; F = 9	54.44 (12.29)
Head Injury	14	M = 11; F = 3	48.25 (15.27)
Anoxia	5	M = 5; F = 0	49.80 (11.05)
Herpes simplex encephalitis (HSE)	6	M = 5; F = 1	42.50 (8.60)
Total	49	M = 36; F = 13	48.75 (11.80)

**Table 2:** Lesion location and apathy diagnosis

***Lesion***	***IAES - 41 score cut off***		
	***Apathy***	***No apathy***	***Total***
Left parietal	3	2	5
Right parietal	2	2	4
Bilateral parietal	0	1	1
Left fronto-temporal	3	4	7
Right fronto-temporal	5	2	7
Bilateral fronto-temporal	14	8	22
*Total (N)*	27	19	46

**Table 3:** Demographic characteristics of participants on the Moral sense, Social awareness, Reality known, Reality unknown, Ekman 60 and Emotion hexagon tests

***Test***	***Participant type***	***Sex***	***Age***
		***M = Male***	***Mean & Std Dev***
		***F = Female***	
Moral sense test	AP = 15	M = 14; F = 1	52.83 (15.03)
N = 42	NA = 8	M = 7; F = 1	58.13 (13.43)
	C = 19	M = 10; F = 9	43.74 (18.63)
Social awareness test	AP = 13	M = 12; F = 1	53.46 (13.31)
N = 50	NA = 12	M = 7; F = 5	56.00 (13.98)
	C = 25	M = 12; F = 13	42.28 (18.11)
Reality Known	AP =11	M = 9; F = 2	53.30 (15.08)
N = 20	NA = 9	M = 8; F = 1	55.33 (14.37)
Reality Unknown	AP = 9	M = 8; F = 1	51.67 (15.02)
N = 21	NA = 12	M = 10; F = 2	55.25 (14.67)
Ekman 60 Test	AP = 21	M = 20; F = 1	58.91 (12.14)
N = 69	NA = 14	M = 9; F = 5	55.82 (15.29)
	C = 34	M = 20; F = 14	51.48 (17.06)
Emotion Hexagon Test	AP = 20	M = 19; F = 1	53.58 (15.89)
N = 60	NA = 13	M = 10; F = 3	57.8 (14.55)
	C = 27	M = 15; F = 15	54.74 (17.89)

*AP*, Patients with Apathy; *NA*, Patients without Apathy; *C*, Healthy controls.

### Materials and procedure

#### Apathy and depressive symptom measures

The informant-rated Apathy Evaluation Scale (AES-I; [66]) was used to assess levels of apathy. The AES is the most widely used apathy scale in both research and clinical practice [67], and one of the few apathy scales with validated psychometric properties [66]. The scale is an 18-item instrument that assesses behavioural, emotional, and cognitive aspects of apathy. Each item on the scale, (e.g. *s/he gets things done during the day*) is rated on a scale of 1 (*Not at all*) to 4 (*A lot*). For a fuller description of this scale, its administration guidelines and psychometric properties see [66]. Patients’ caregivers provided the ratings on the scale. A higher score on the scale indicates more apathy. An AES-1 score of 41 and above was taken as indicative of the presence of apathy, in line with the supplementary scoring guidelines obtained from the scale’s authors. The level and presence of depressive symptoms were evaluated using the Beck Depression Inventory (BDI; [68]). We classified scores at or above the score of 11 as indicative of the presence of depression.

### Executive function tests

#### The Brixton test

The Brixton Spatial Anticipation test [69] provided a measure of cognitive control for our patients. We chose this test because of its limited semantic loading, and also because research shows it is robust enough and sensitive to a number of executive processes, including set shifting and rule or feedback use in acquired brain damage [69,70]. The test consists of a series of pages in a booklet. Each page has the same basic design with ten circles in two rows of five each, one of which is coloured blue. The participants’ task is to detect patterns in the sequence of the blue circle and then predict its position on subsequent pages. Further executive function measures were also obtained on the Stroop [71] and Hayling [69] tests.

#### The Stroop test

On the Stroop test participants name the colour of printed colour-words as fast as they can. A successful performance on the test requires the inhibition of the tendency to read the colour-words.

#### The Hayling test

On the Hayling test participants are read incomplete sentences that they should complete with a word that is not related in any way to the incomplete sentence. Successful performance on this test requires the inhibition of a pre-potent tendency to complete sentences.

### Social cognition tests

#### Moral sense test

The moral dilemma scenarios used to assess moral reasoning on the test were taken from the classical ‘trolley dilemmas’ used to explore intuitions about the permissibility of sacrificing the well-being of one person in order to save many others [72]. Participants were asked to rate on a 1–7 likert-type scale the moral permissibility of either intentionally harming one person in order to save many others (intended harm), or letting some foreseeable harm happen to one person in order to save many more (foreseen harm). The test consisted of 30 story vignettes made up of 10 control items, 10 intentional harm items, and 10 foreseeable harm items (see Additional file 1: Box 1).

#### Social awareness test

Dewey [73]’s Social Awareness Test (SAT) was used to assess participants’ judgements of appropriateness of behaviour. The SAT consists of 9 short stories describing ordinary and unusual social interactions which participants have to judge by choosing (A) if they think the behaviour is *“Fairly normal behaviour in that situation”*, (B) if they think the behaviour is *“strange behaviour in that situation”*, (C) if they judge the behaviour as *“very eccentric behaviour in that situation”* and (D) if they think the behaviour described is *“shocking behaviour in that situation”* (see Additional file 2: Box 2).

#### Theory of mind tests

Two ToM tests were used: the *Reality Known* and the *Reality Unknown* tests. The tests comprised a series of carefully constructed non-verbal belief reasoning videos designed to control for deficits in memory, executive function and comprehension. See [74] for a full description of the tests. For example, in the *Reality-Unknown* test, a man placed an object in one of two identical boxes in full view of another female actor but without the participant seeing in which box the object was placed (participants did not know the reality about which box contained the object). The woman then left the room, and while away, the male actor swapped the two boxes. Upon returning, the woman pointed at one of the boxes. Locating the box containing the object then required participants to infer on the woman’s false belief about the two boxes and to realize that the object was therefore in the box other than the one the woman pointed at. This ensured participants’ attribution of false belief could not be disrupted by their own knowledge of the correct answer. To correctly infer the woman’s false belief, participants did not need to inhibit their own self perspective. Deficits related to inhibiting one’s self perspective are quite common in patients with brain damage, and have been shown to confound performance on false belief ToM tasks [75]. Inhibition control trials thus followed the same sequence as false belief trials, but instead of swapping the boxes, the man performed a visible transfer of the object from one box to the other in full view of the participant. Other trials in the two tests controlled for memory capacities. Working memory control trials followed the same sequence as false belief trials except that the woman indicated one of the boxes before leaving the room. This enabled the participant to infer the location of the object. In this case, inferring the correct location of the object did not require the attribution of a false belief, but as in the false-belief trial, participants had to remember that the location of the object changes when the boxes are swapped and had to maintain this information until a response was requested. The tasks’ limited use of verbal language also controlled for problems related to semantic loading, which is a problem with most story-based false belief ToM tasks [76]. In the *Reality Known* test, the participants were aware of the location of the object.

#### Emotion perception tests

The *Ekman 60* and the *Emotion Hexagon* tests from the Facial Expressions of Emotions Stimuli and Tests (FEEST) CD-ROM [77] were used to assess perception of facial expressions of emotion. Both tests assess perception of the facial expressions of six basic emotional states of anger, disgust, fear, happiness, sadness, and surprise. Pictures of the emotional faces are presented on a computer screen.

On the Ekman 60 test each emotion is represented on 10 face images, giving a total of 60 images for the entire test. The faces on the emotion hexagon test are computer-manipulated and morphed such that each emotion is presented in graded levels of difficulty and hence the emotions are comparatively more difficult to recognise than those on the Ekman 60 test. Each emotion is mixed or blended with another which it is most likely confused with, resulting in the following sequence of mixes; happiness-surprise-fear-sadness-disgust-anger. For example, the happiness-surprise continuum is morphed such that happiness would be blended with surprise (in ratios of 90% happiness with 10% surprise; 70% happiness with 30% surprise; 50% happiness with 50% surprise; 30% happiness with 70% surprise; 10% happiness with 90% surprise). The ends of this sequence (anger and happiness) join to form the emotion hexagon (see [77], for a detailed description of how the emotion hexagon test stimulus was made). The Emotion hexagon test has a total of 120 trials split into 4 blocks. In both the Ekman 60 and the Emotion hexagon tests, participants responded by using a mouse to click on one of the 6 emotion words (Anger, Disgust, Fear, Sadness, Surprise, and Happiness) presented at the bottom of the screen.

### Summary on tests

Table 3 gives the participant composition on each of the tests used in this study. For a variety of reasons, ranging from patient drop out from the study to individual patients’ specific neurocognitive impairments which made them unsuitable for some tests, it was not possible to assess all patients on every task. Normal participants were not assessed for depression and apathy symptoms because a history of psychiatric illness was part of our exclusion criteria. Neither did we test normal participants for executive deficits because we didn’t have any practical or theoretical reason to expect them to present with these deficits.

### Data analysis

Exploratory data analysis was done to test for normality of distribution and equality of variance. Kolmogorov-Smirnov (K-S) tests for normality of distribution and Levene’s test for equality of variance where performed to ascertain whether data met assumptions for the use of parametric tests. Non-parametric test equivalents were performed on data that violated these assumptions. The correlation coefficient (r) was used as a measure of effect size.

## Results

### Apathy and depression

Twenty five patients (51%) met the criteria for the presence of apathy in the absence of depression. The mean score for apathy in the group of patients with apathy symptoms was 50.96 (SD = 9.64) and that for patients without apathy was 37.5 (SD = 7.57). One patient (2%) met the criteria for depression but not apathy. Two patients (4.1%) met the criteria for the presence of both apathy and depression. As groups, the mean depression score for patients with apathy was 11.96 (SD = 7.97) and 11.83 (SD = 4.73) for patients without apathy. Twenty one patients (42.9%) did not have either apathy or depression.

### Executive functions

#### Brixton test

Brixton scores for patients with apathy symptoms (M = 27.72, SE = 2.11) were not significantly different from those of patients who had no significant apathy symptoms (M = 24.31, SE = 1.98; t (22) = 1.14, p > 0.05; which represented a small sized effect, r = .27. In subsequent analyses, we also included the Brixton score as a covariate because this test has been shown to demonstrate sensitivity to a variety of executive deficits, such as perseverative behaviour and deficits in feedback use.

#### Hayling test

No significant differences were found between patients with apathy (Mdn = 20) and those without apathy (Mdn = 11), Z = 1.10, ns, r = 0.20 on Section 1 of the Hayling test. Also, no significant difference were found between patients with apathy (Mdn = 33) and those without apathy (Mdn = 12), Z = .91, ns, r = .17 on Section 2 of the Hayling test.

#### Stroop test

Patients with apathy (Mdn = 97) did not significantly differ from those without apathy on Section 1 of the stroop test (Mdn = 95.5), Z = 0.552, ns, r = .12. T-test comparisons on Section 2 of the stroop test also showed no significant differences in performance between patients with apathy (M = 50.13, SE = 8.74) and those without apathy (M = 45.00, SE = 11.35; t (21) = .35, p > 0.05, which represented a small sized effect r = .08*.*

### Social cognition

#### Moral sense test

We excluded from analysis all subjects whose responses fell more than 2 standard deviations from the control mean on more than 2 of the 10 control scenarios because we felt such participants did not understand the stories. For this reason, data for 9 patients were excluded from analysis. Data for 2 of the normal participants were also excluded because they failed to complete the test. Table 4 shows the means and standard deviations for the three participant groups on the MST foreseen and intended harm dimensions.

**Table 4:** Means and standard deviations for responses to ‘Intended harm’ and ‘Foreseen harm’ moral dilemmas of the Moral sense test

	***Control (n = 17)***		***Apathy (n = 7)***		***No apathy (n = 7)***	
**Harm**	***Mean***	***Std Dev***	***Mean***	***Std Dev***	***Mean***	***Std Dev***
Intended	2.14	0.94	4.63	1.52	2.91	1.23
Foreseen	3.06	0.85	4.63	1.30	3.82	1.41

#### Intended harm

A one way ANOVA showed that the 3 participant groups differed on how they judged intentional harm F(2, 28) = 11.55, p < 0.001. Tukey HSD post hoc tests revealed that patients with apathy judged that intentionally harming one person to save many more as more permissible (Mean = 4.63) than healthy controls (Mean = 2.14, p < 0.001), and patients without apathy (Mean = 2.91, p < 0.05). There were no significant differences in judgements between healthy controls and patients without apathy (p > 0.05). Including Brixton scores as a covariate while comparing patients with apathy and those without apathy’s intentional harm scores showed no significant effects of the Brixton score on moral judgements, F(1,11) = 2.17, ns., and the differences in moral judgements between the 2 patient groups also remained significant, F(1,11) = 7.69, p < 0.05. Including the BDI score as a covariate showed a borderline effect of depression and moral judgements (F(1, 11) = 5.35, p = 0.05) but the difference between moral judgements in patients with apathy and those without apathy remained significant (F(1, 11) = 11.23, p < 0.01).

#### Foreseen harm

Judgements on the foreseen harm dimension differed across the 3 participant groups, F(2, 27) = 6.82, p < 0.01. Post hoc Tukey HSD tests showed no differences on judgements on the moral permissibility of allowing some foreseen harm to happen to one person in order to save others between patients with apathy (Mean = 4.90) and those without apathy (Mean = 3.82, p > 0.05). There were significant differences between healthy control participants (Mean = 3.06) and patients with apathy (p < 0.01) but no significant differences between healthy controls and patients without apathy (p > 0.05). Including Brixton scores as a covariate while comparing patients with apathy against scores for those without apathy showed no effect of the Brixton score on moral judgements (F(1,11) = 2.91, ns) though the differences in moral judgements between patients with apathy and those without apathy became borderline significant (F(1,11) = 4.27, p < .01). There was no significant effect of the BDI score on moral judgements, F(1,11) = 1.25, ns, and the difference between patients with apathy and those without apathy’s foreseen harm judgements remained non-significant F(1,11) = 2.98, ns.

### Social awareness test

We took “A” responses on the test to indicate that participants judged protagonists’ behaviour as normative, while “B”, “C”, and “D” responses indicated that participants judged the behaviour as a social norm violation. Healthy controls (see Table 3) provided normative data. Only items on which we obtained more than 95% consensus (p < .05) from healthy controls were used for this analysis. This consensus was obtained on 12 out of the 23 SAT test items. On these 12 items more than 95% of the controls identified 5 behaviours as normal, and 7 as norm violations.

On the basis of our normative data, relatively fewer patients with apathy (84.6%) correctly identified normal behaviour compared to those without apathy (91.7%). Chi-square tests revealed that the percentage of those who correctly identified normative behaviour differed according to the presence of apathy symptoms (χ^2^[3] = 200, p < .001). Based on the odds ratio, patients without apathy were 2.03 times more likely to correctly judge normal behaviour as normal than patients with apathy symptoms. Also, fewer patients with apathy (Mean = 78%) correctly identified norm violations compared to those without apathy (89.3%). Chi-square tests revealed that the percentage of those who correctly identified norm violations differed according to the presence of apathy symptoms (χ^2^ (3) = 200, p < .001). Based on the odds ratio, patients without apathy were 2.28 times more likely to correctly identify social norm violations compared to patients with apathy.

When patients who took the SAT test were compared on executive functioning and depressive symptoms, there were no significant differences between those with apathy symptoms and those without apathy on the Brixton Spatial Anticipation test (apathy patients median = 27; non-apathy patients median = 21; U = 40.5, ns). There were also no significant differences in depressive symptoms levels on the BDI between patients with apathy (Median = 17) and those without apathy (Median = 11.5; U = 52.5, ns). The significant differences in social awareness on the SAT cannot therefore be explained by differences in executive function or depressive symptoms levels.

### Theory of mind tests

We did not include a normal control group for the ToM tests used in this study because a pilot study on the tests showed that people without neurological damage performed at ceiling level. See also [74]. We tested the effects of apathy symptoms on ToM by comparing performance between patients with and without apathy on the false belief score and on control items for working memory, inhibition, true belief and the filler scores for both the reality known and reality unknown tasks.

#### Reality known test

A one way MANOVA comparing patients with and without apathy on false belief, working memory, anti-strategy, and filler scores on the Reality known test revealed non-significant main effects for apathy, Wilk’s λ = .853, F(4,15) = .646, p > .05 partial eta squared .147. Power to detect the effect was .166 (see Table 5 for the full results).

**Table 5:** Statistical comparisons on Theory of mind scores for patients with apathy versus those without apathy

	**Apathy**	**No apathy**	**Manova statistic**
*Reality Known Task*	9	8	
False Belief task			
Memory Control	12	11	P > .05
Anti-strategy control	12	12	
Filler trials	12	12	
*Reality Unknown Task*	*Mean*	*Mean*	
False Belief task	7.56	7.42	
Working Memory	10.11	9.83	P > .05
Inhibition Control	12	12	
True Belief	10.11	9.83	
Fillers	12	11.5	

#### Reality unknown test

A one way Manova comparing patients with and without apathy on false belief, working memory, inhibition, true belief (comprehension) and filler scores of the Reality unknown test revealed non-significant effects for apathy, Wilk’s λ = .969, (4, 16) = .128, p > .05 partial eta squared .031. Power to detect the effect was .07 (see Table 5 for the full results).

### Emotion Perception tests

#### Ekman 60 test

Kruskal-Wallis tests comparing the total number of correctly identified facial expressions of emotions across the three participant groups showed a significant effect of participant group on emotion recognition H(2) = 34.82, p < 0.001. Mann–Whitney post hoc tests with a Bonferroni correction of 0.0167 significance level showed that the healthy controls (*Mdn* = 51) correctly recognised more facial expressions of emotions compared to both patients with apathy (*Mdn* = 41; U = 52.5, p < 0.001, r = −.71) and those without apathy (*Mdn* = 51; U = 51.5, p < 0.001, r = −.61). The difference between scores for patients with apathy and those without apathy was not significant (U = 140.5, ns, r = −.04). Comparisons on each individual emotion across the three groups also yielded the same pattern of results. Due to the loss of power likely to result from these multiple comparisons across individual emotion categories, a Bonferroni correction was applied (see Table 6 for the full results).

**Table 6:** Statistical comparisons between patients with apathy, patients without apathy, and normal controls on tests for the perception of facial expressions of emotion

**Emotion**	**Kruskal-Walis test result**	**Post hoc comparisons**
		**Bonferroni correction = 0.0167**
**EKMAN 60**	H(2) = 34.82, p < 0.001	C vs A U = 52.5, p < 0.001, r = −.71*
		C vs NA U = 51.5, p < 0.001, r = −.61*
		A vs NA U = 140.5, ns, r = −.04
Anger	H(2) = 17.95, p < 0.001	C vs A U = 165, p < 0.001, r = −.46*
		C vs NA U = 84, p < 0.001, r = −.51*
		A vs NA U = 134, ns, r = −.08
Disgust	H(2) = 22.71, p < 0.001	C vs A U = 127.5, p < 0.001, r = −.55*
		C vs NA U = 78.5, p < 0.001, r = − .51*
		A vs NA U = 125.5, ns, r = −.12
Fear	H(2) = 16.78, p < 0.001	C vs A U = 143.5, p < 0.001, r = −.5*
		C vs NA U = 170, p < 0.01, r = −.46*
		A vs NA U = 126, ns, r = −.12
Happiness	H(2) = 9.21, p < 0.05	C vs A U = 289, p < 0.01, r = −.35*
		C vs NA U = 170, p < 0.01, r = −.46*
		A vs NA U = 136, ns, r = −.09
Sadness	H(2) = 15,47, p < 0.001	C vs A U = 213, p = 0.01, r = −.35*
		C vs NA U = 87.5, p < 0.001, r = −.51*
		A vs NA U = 92.5, ns, r = .32
Surprise	H(2) = 9.27, p < 0.01.	C vs A U = 208.5, p < 0.01, r = −.35*
		C vs NA U = 137, p < 0.01, r = −.34*
		A vs NA U = 146, ns, r = −.001
**EMOTION HEXAGON**	H(2) = 26.11, p < 0.001	C vs A U = 45, p < 0.01, r = −.71*
		C vs NA U = 57.5, p < 0.01, r = −.54*
		A vs NA U = 127, ns, r = −.02
Anger	H(2) = 26.11, p < 0.001	C vs A U = 97, p < 0.01, r = −.55*
		C vs NA U = 71, p < 0.01, r = −.48*
		A vs NA U = 130, ns, r = 0
Disgust	H(2) = 8.22, p < 0.05	C vs A U = 116, ns, r = −.09
		C vs NA U = 92.5, p = 0.015, r = − .39*
		A vs NA U = 116, ns, r = −.09
Fear	H(2) = 23.06, p < 0.001	C vs A U = 67, p < 0.01, r = −.64*
		C vs NA U = 59.5, p < 0.01, r = −.53*
		A vs NA U = 108, ns, r = −.14
Happiness	H(2) = 9.38, p < 0.01	C vs A U = 160, p < 0.01, r = −.44*
		C vs NA U = 143, ns, r = −.22
		A vs NA U = 95, ns, r = −.25
Sadness	H(2) = 27.39, p < 0.001	C vs A U = 72, p < 0.01, r = −.65*
		C vs NA U = 31.5, p < 0.01, r = −.69*
		A vs NA U = 107, ns, r = −.15
Surprise	H(2) = 6.69, p < 0.05	C vs A U = 167, ns, r = −.33
		C vs NA U = 107, p < 0.01, r = −.32*
		A vs NA U = 120, ns, r = −.06

*A*, Patients with Apathy; *NA*, Patients without Apathy; *C*, Healthy controls.

*Between groups difference is significant.

#### Emotion hexagon test

Kruskal Wallis tests showed an overall reliable difference between the three groups of participants in their ability to recognise emotions, H (2) = 26.11, p < 0.001. Post Hoc Mann Whitney tests showed that healthy control participants (*Mdn* = 110) recognised significantly more expressions compared to both patients with apathy (*Mdn* = 87; U = 45, p < 0.01, r = −.71) and without (*Mdn* = 75; U = 57.5, p < 0.01, r = −.54). There were no significant differences between patients with apathy and those without apathy (U = 127, ns, r = −.02). Table 6 shows the full results across individual emotion categories. To address possible power slippages due to multiple test comparisons, a Bonferroni correction was applied.

## Discussion

### Overview

Our results provide new evidence that apathy symptoms in patients with acquired brain damage are associated with making impaired social judgements. For instance, patients with apathy tended to judge that intentionally harming one person (intended harm) in order to save many more (as in pushing a bulky man onto a runaway trolley in order to stop it from hitting 5 others) was morally permissible, while patients without apathy and healthy controls both tended to judge such means-to-an-end intentional harm as not permissible. On the other hand, where harm to one person was not directly intended, but a foreseeable side effect of diverting the harm from five people (foreseen harm), moral judgements for both patients with apathy and those without apathy were not significantly different. Furthermore, patients with apathy failed to recognise as many instances of norm violations as patients without apathy on the SAT, while also misjudging more normal behaviours as norm violations. The majority of our patients with apathy symptoms also had frontal lesions, confirming findings from other studies on the association between frontal damage and both socio-cognitive deficits and apathy symptoms (see discussion below). Performance scores on social cognition measures for emotion perception (Ekman 60 and Emotion hexagon tests), and ToM failed to separate patients with apathy from those without apathy symptoms, though the patients with apathy tended to perform worse. In these cases, the patients as a whole were reliably worse than controls.

### Apathy and moral reasoning

The current data on our Moral sense test that highlights changes in moral reasoning in patients with apathy may be accounted for in a variety of ways. For example, recent research suggests a crucial role of emotional influences on moral reasoning. It has been demonstrated that in moral dilemmas where harm is both intentional and direct, an emotionally aversive reaction is generated that makes people disapprove of the act [64,78]. Valdesolo and DeSteno [64] further found that inducing positive emotions (to counteract the aversive emotional responses involved in intentional harm dilemmas) made normal participants more likely to approve the harm. More evidence for the role of emotional processes in social behaviour has been documented by Bechara et al. [56], who demonstrated that patients with prefrontal damage my fail to generate emotion signals that help bias behaviour towards adaptive social acts. See also [61,79]. The responses of the patients with apathy here then may reflect a lack of emotional engagement. The most salient feature of apathy involves attenuated emotional behaviour [17].

In support of the above suggestions, Mendez, Anderson, and Shapira [80] found that emotionally blunted patients with frontotemporal dementia were also disproportionately more likely to give utilitarian responses in response to moral dilemmas similar to those used in this study. It should also be noted that damage to brain areas thought to subserve this emotional input, such as the anterior cingulate cortex and the ventro-medial pre-frontal cortex (VMPC) [81,82] have also been consistently associated with the presence of apathy [2,83,84]. Our current results are consistent with this explanation since the majority of those patients who had apathy symptoms and also malperformed on the moral sense test (57.1%) had bilateral prefrontal lesions. In this context, the evidence suggests that an underlying affective processing deficit might underlie apathy symptoms. Also in support of this position, Levy and Dubois [21] argue that lesions to the orbital-medial prefrontal regions can disrupt affective processing of the emotional signals that are responsible not only for directing ongoing or forthcoming behavior, but that also play a role in decoding the context and motivational value of behavioural events. Such disruptions then make it difficult for patients to elaborate or formulate action plans, leading to apathetic behaviour.

Our study is however limited in the extent to which it can attribute poor performance on the moral dilemmas to emotional deficits such as those proposed by Damasio et al. [82] since measures of emotional responding were not concurrently taken. It is also important to note that other researchers working with different clinical samples have explained the type of moral reasoning deficits found in our patients in terms of impaired theory of mind processing for moral judgements [85]. However ToM performance did not distinguish patients with apathy from those without apathy in this study (see below).

### Apathy and social awareness

Patients with apathy were also significantly impaired on the Social awareness test where they were required to judge a protagonist’s behaviour in a story vignette by ascertaining whether an individual had violated socio-conventional norms or whether the behavior was normal. The relatively poorer performance by these patients on this test included rating common social behaviours as ‘shocking’ or ‘eccentric’ while unusual or inappropriate social behaviours were sometimes judged as normal. Thus the performance of these patients cannot simply be attributed to a response bias or a lower level of response. It is possible that there are emotional contributions to social behaviour judgements akin to those linked to moral judgements, but this has not been established. In the context of autistic spectrum disorders it has been argued that impairments on the Social awareness test reflect reduced processing of the social context of a situation, an inability to take the perspective of the protagonist in the story, or are a result of a rigid application of social rules [73]. We can with some confidence rule out social perspective-taking (ToM) deficits as critical, given that the patients with apathy and those without apathy did not differ in this capacity. Further work is required to establish the factors that generate our observed dissociation.

### Apathy and theory of mind

Our results suggest that deficits in ToM may not be enough to account for apathy symptoms. Our results on ToM differ from studies that have reported an association between ToM deficits and related negative symptom spectrum disorders which also include apathy symptoms [43,86]. There could be a number of reasons to explain the findings. For instance, there is disagreement about how best to conceptualise ToM processes, ranging from the critical role of belief reasoning [48] to the recognition of mental states such as emotions [87]. Consequently, different sets of ToM tests have been used, sometimes on different clinical samples. Some of the ToM tests also fail to control for the influence of other ancillary processes that are separate to the ToM process but are recruited during task performance, such as executive functioning, comprehension, and memory. The tasks used in this study employed more robust mechanisms to isolate ToM processes from these potential confounding variables (e.g., the need for a participant to inhibit their own self-knowledge).

### Apathy and emotion perception

Patients with high levels of apathy did not differ from those without symptoms of apathy. Compared to normal controls, both patient groups showed significant emotion recognition deficits. One possible reason for this lack of differential performance between the two patient groups could be that the recognition of emotion from facial expressions is dependent on diverse processes, mediated by different brain areas. Damage to any of these areas could produce impairments on emotion recognition tests, for a variety of reasons. For example, according to Adolphs [88], the occipitotemporal, orbitofrontal, and somatosensory cortices, together with the hippocampal formation, hypothalamus, and brainstem all play a significant role in the processes involved in extracting and labelling emotion-related information from facial expressions.

## Conclusion

Our study suggests that patients with brain damage and apathy symptoms can have significant problems knowing what is or is not appropriate social conduct. The social impairments shown by these patients may in part reflect socio-cognitive deficits some of which relate to judging the appropriateness or inappropriateness of behaviour in complex interpersonal settings. The deficits are not linked to depression, impaired executive functions or ToM deficits in those individuals with apathy, but they may relate to particular aspects of apathy symptoms like social withdrawal, incapacity to show event-appropriate behavioural and emotional responses, and a failure to meet social expectations.

This study provides the first attempt to develop a socio-cognitive phenotype of apathy symptoms. Such a profile can be of great diagnostic and rehabilitation value [89,90], and is important in improving our understanding of the socio-cognitive correlates of apathy.

### Strength and weaknesses

One main weakness in this study is the lower patient numbers on some of the tests, especially the moral sense test. This might have contributed to inadequate power since non-significant findings on tests such as the Ekman test were often in the expected direction. In other words, there were large differences between healthy controls and patients without apathy on one side and those with apathy on the other. Larger sample sizes might probably have produced more significant results. Future studies may consider using larger samples of patients and also using shorter socio-cognitive tests with less language loading.

## Competing interests

There are no financial or non-financial competing interests on this study.

## Authors’ contributions

PN carried out the study, analysed the data and produced the draft. GH and SD improved on the draft and also helped with the recruitment of patients. All authors read and approved the final manuscript.

## Pre-publication history

The pre-publication history for this paper can be accessed here:

http://www.biomedcentral.com/1471-2377/14/18/prepub

## Supplementary Material

Additional file 1 The Moral Sense Test: sample item. Click here for file

Additional file 2 The SAT test sample item. Click here for file
