# Supplementary material for: Exploring social cognition in patients with apathy following acquired brain damage
Source: BMC Neurol. 2014 Jan 23;14:18. doi: 10.1186/1471-2377-14-18 (PMC3943587; doi:10.1186/1471-2377-14-18)
Supplement: Additional file 1 — The Moral Sense Test: sample item. [file 1471-2377-14-18-S1.doc]

**Box 1**

**The Moral Sense Test: sample items.**

**Ben (control item)**

Ben notices an empty boxcar rolling out of control. It is moving so fast that anyone it hits will die. The boxcar is headed down an empty track. Standing on a side track is one person. Ben is standing next to a switch that can be flipped to turn the boxcar down the side track. If Ben does not flip the switch, the boxcar will continue down the empty track, leaving the one person safe. If Ben flips the switch, the boxcar will turn down the side track and hit the one person.

Ben flips the switch. The boxcar turns down the side track and hits the one person.

Flipping the switch was:

0 0 0 0 0 0 0

Forbidden Permissible Obligatory

**Emily (Foreseeable Harm)**

Emily notices an empty boxcar rolling out of control. It is moving so fast that anyone it hits will die. The boxcar is headed down the track toward five people. Emily can flip a switch, turning the boxcar away from the five onto the side track. However, there is one person on the side track. If Emily flips the switch, the boxcar will hit the one person on the side track, but the five will be saved. If Emily does not flip the switch, the one person will be safe, nut the five will be hit.

Emily flips the switch. The one person is hit, but the five are saved. Flipping the switch was:

0 0 0 0 0 0 0

Forbidden Permissible Obligatory

**Colin (Intended Harm)**

On a footbridge above the railroad tracks, Colin watches an empty, out of control boxcar about to hit five people. Colin can stop the boxcar by dropping a very heavy weight into its path. But the only heavy weight around is a heavy person next to him also watching the boxcar from the footbridge. If Colin pushes the person onto the track, the boxcar will hit the person, but the person’s body will stop the boxcar from hitting the five. If Colin does not push the person, the person will be safe, but the boxcar will hit the five others.

Colin pushes the person onto the tracks. The person dies, but the five others are saved. Pushing the person onto the tracks was:

0 0 0 0 0 0 0

Forbidden Permissible Ob
