# Supplementary material for: Exploring social cognition in patients with apathy following acquired brain damage
Source: BMC Neurol. 2014 Jan 23;14:18. doi: 10.1186/1471-2377-14-18 (PMC3943587; doi:10.1186/1471-2377-14-18)
Supplement: Additional file 2 — The SAT test sample item. [file 1471-2377-14-18-S2.doc]

**Box 2**

**The SAT test sample item**

**The forgotten name.**

Paul, 33, had a small shop where he renovated old furniture. Sometimes a customer would ask to have some work done in her home. On one such occasion an elderly lady called him to stain a scratch on her desk. *Unfortunately, Paul forgot to jot down her name when he wrote the address*. The lady greeted her warmly at her door, saying, “Come right in, Paul. I have heard that your work is good”. Ashamed because he had forgotten her name, *Paul waited until she left the room and peeked into a drawer*. Sure enough, he found some letters addressed to Mrs. Isabel DeWitt, and this jogged his memory. Satisfied, Paul shut the drawer without disturbing anything and soon he had the scratch nicely finished. When the lady of the house saw it she said, “That’s perfect! How much do I owe you, Paul?” *He replied, “It did not take very long, so £5 will be fine, Isabel.”*
